# Vascular Response on a Novel Fibrin-Based Coated Flow Diverter

**DOI:** 10.1007/s00270-021-03007-9

**Published:** 2021-12-16

**Authors:** Ruben Mühl-Benninghaus, Frederik Fries, Mara Kießling, Toshiki Tomori, Stefanie Krajewski, Andreas Simgen, Sabina Bauer, Natascha Hey, Eduard Brynda, Johanka Taborska, Tomáš Riedel, Wolfgang Reith, Giorgio Cattaneo, Christoph Brochhausen

**Affiliations:** 1 Department of Neuroradiology, Saarland University Hospital, Kirrberger Strasse, 66424 Homburg, Germany; 2 Institute of Pathology, University of Regensburg, Regensburg, Germany; 3 Department of Thoracic, Cardiac and Vascular Surgery, University Hospital Tuebingen, Tübingen, Germany; 4 Acandis, Pforzheim, Germany; 5 Institute of Macromolecular Chemistry, Czech Academy of Sciences, Prague, Czech Republic; 6 Institute for Biomedical Engineering, University of Stuttgart, Stuttgart, Germany

**Keywords:** Flow diverter, Coating, Surface modification, Biocompatibility

## Abstract

**Purpose:**

Due to thromboembolic complications and in-stent-stenosis after flow diverter (FD) treatment, the long-term use of dual antiplatelet treatment (DAPT) is mandatory. The tested nano-coating has been shown to reduce material thrombogenicity and promote endothelial cell proliferation in vitro. We compared the biocompatibility of coated (Derivo Heal) and non-coated (Derivo bare) FDs with DAPT in an animal model.

**Methods:**

Derivo® bare (*n* = 10) and Derivo® Heal (*n* = 10) FD were implanted in the common carotid arteries (CCAs) of New Zealand white rabbits. One additional FD, alternately a Derivo bare (*n* = 5) or Derivo Heal (*n* = 5), was implanted in the abdominal aorta (AA) for assessment of the patency of branch arteries. Histopathological examinations were performed after 28 days. Angiography was performed before and after FD implantation and at follow-up.

**Results:**

Statistical analysis of the included specimens showed complete endothelialization of all FDs with no significant differences in neointima thickness between Derivo® bare and Derivo® Heal (CCA: *p* = 0.91; AA: *p* = 0.59). A significantly reduced number of macrophages in the vessel wall of the Derivo Heal was observed for the CCA (*p* = 0.02), and significantly reduced fibrin and platelet deposition on the surface of the Derivo Heal was observed for the AA. All branch arteries of the stented aorta remained patent.

**Conclusion:**

In this animal model, the novel fibrin-based coated FD showed a similar blood and tissue compatibility as the non-coated FD.

## Introduction

Flow diversion has revolutionized the treatment of complex and broad-necked intracranial aneurysms [1]. Recent clinical data demonstrated superior occlusion and less reperfusion of aneurysms treated with flow diverters (FDs) compared to both coiling and stent-assisted coiling [2]. However, FD implantation carries the risks of periprocedural and delayed complications such as ischemia due to thromboembolic events [3, 4] or the development of in-stent stenosis [5, 6]. Dual anti-platelet therapy (DAPT) is mandatory to decrease these risks [7]. For acutely ruptured aneurysms, when surgical clipping and coiling alone is not feasible, there is an unmet need for stents or even additional thrombogenic implants such as FDs under reduced DAPT or single antiplatelet therapy (SAPT). Thus, a current focus of research lies on the development of new surface technologies for neurovascular FDs with the goal of reducing the surface thrombogenicity of these devices [8]. Surface alterations of the stents like electropolishing and annealing (e.g., BlueXide®, Acandis Pforzheim, Germany) are approaches to reduce foreign body reactions. Apart from heparin coating [9], different biocompatible polymer coatings are currently available for neurovascular devices. The phosphatidylcholine coating called Shield Technology™ (Medtronic, Irvine, CA) was developed to inhibit platelet adhesion [10]. Another coating, a glycan-based polymer coating called pHPC® (Phenox, Bochum, Germany) was developed with hydrophilic properties that reduce thrombocyte adhesion [11–13].

The aim of this in vivo study was to assess the blood and tissue compatibility of a newly developed fibrin-based nano-coating (Heal coating; Acandis, Pforzheim, Germany) for FDs using a rabbit model, paying particular attention to neointima formation.

## Materials and Methods

### FD Design

The Derivo Embolization Device (DED; Acandis, Pforzheim, Germany) is a radiopaque self-expanding braided nitinol FD. It consists of 48 nitinol crossing wires and has closed distal ends and a flaring of 25° to the outside at both ends for secure wall apposition. The device is connected to a transport wire into an introducer.

### Modification of FDs with a Fibrin-based Nano-Coating

Fibrin-based nano-coating is a novel surface modification technology in which fibrinogen molecules are converted to fibrin. The result is a completely cured fibrin network, i.e., it is inert in terms of coagulation, because it mimics the final step of hemostasis. After production of the bare device, the coating is applied to the final stent product. FDs were coated with the fibrin mesh using a modified step-by-step technique as described previously [14]. The coating completely covers the FD struts on both the in- and outside over the complete circumference and length. The fibrin mesh is functionalized using covalently attached heparin. After coating, characterization of fibrin amount and heparin activity was performed, and thickness was measured on selected wires by atomic force microscopy (data not shown).

### Animal Experiments

All experiments were approved by the institutional animal care and use committee (No 07/17). The animal research reported in this study was conducted in accordance with the ARRIVE guidelines. A rabbit model was chosen, because the supra-aortic vessels of these animals have a similar diameter compared to the intracranial arteries of humans. Furthermore, it is the most widely used model to assess neurovascular devices prior to human use [15].

Ten New Zealand white rabbits received DAPT therapy with aspirin (ASA) and clopidogrel (10 mg/kg/day), given orally starting from three days prior to FD implantation until the angiography and sacrifice at 28 days. Previous studies have shown that neointima growth and reactions of the vessel walls, especially in the rabbit model, are at a maximum after about 3–4 weeks [16–18]. A total of 30 FDs were implanted in 10 rabbits, 3 per rabbit: 1 non-coated FD (Derivo bare) and 1 coated FD (Derivo Heal) were implanted in either the left or right common carotid artery (CCA) with the side chosen at random and in a blinded manner; in addition, each animal received either a Derivo bare (*n* = 5) or a Derivo Heal (*n* = 5) implant in the abdominal aorta (AA).

### Flow Diverter Placement

All procedures were performed under general anesthesia with intramuscular ketamine (60 mg/kg)/Rompun® 2% (6 mg/kg) and maintenance with ketamine (60 mg/kg)/Rompun 2% (6 mg/kg) in 10 ml NaCl at a flow rate of 2.5 ml/h via an ear vein. The right femoral artery was surgically exposed, and a 5 Fr sheath was inserted. A dose of 300 U heparin was administered intravenously during the procedure. Angiography of the CCAs and the AAs was performed using a 4 Fr diagnostic catheter (Glidecath, Terumo Europe, Leuven, Belgium). A 0.027-inch Headway® microcatheter (MicroVention Inc., Aliso Viejo, USA) with a 0.014-inch Synchro® microwire (Stryker, Kalamazoo, USA) was used to access the CCAs and the AAs for the deployment of the bare and coated FDs. The size of the implanted FDs was 3.5 × 15 mm for CCAs and 4.5 × 20 mm for AAs. The devices were oversized to ensure adherence to the vessel wall. After angiographic control (described below), all endovascular materials were removed, the femoral arteries ligated, and the wounds sewn.

### Angiographic Imaging

**Fig. 1:**
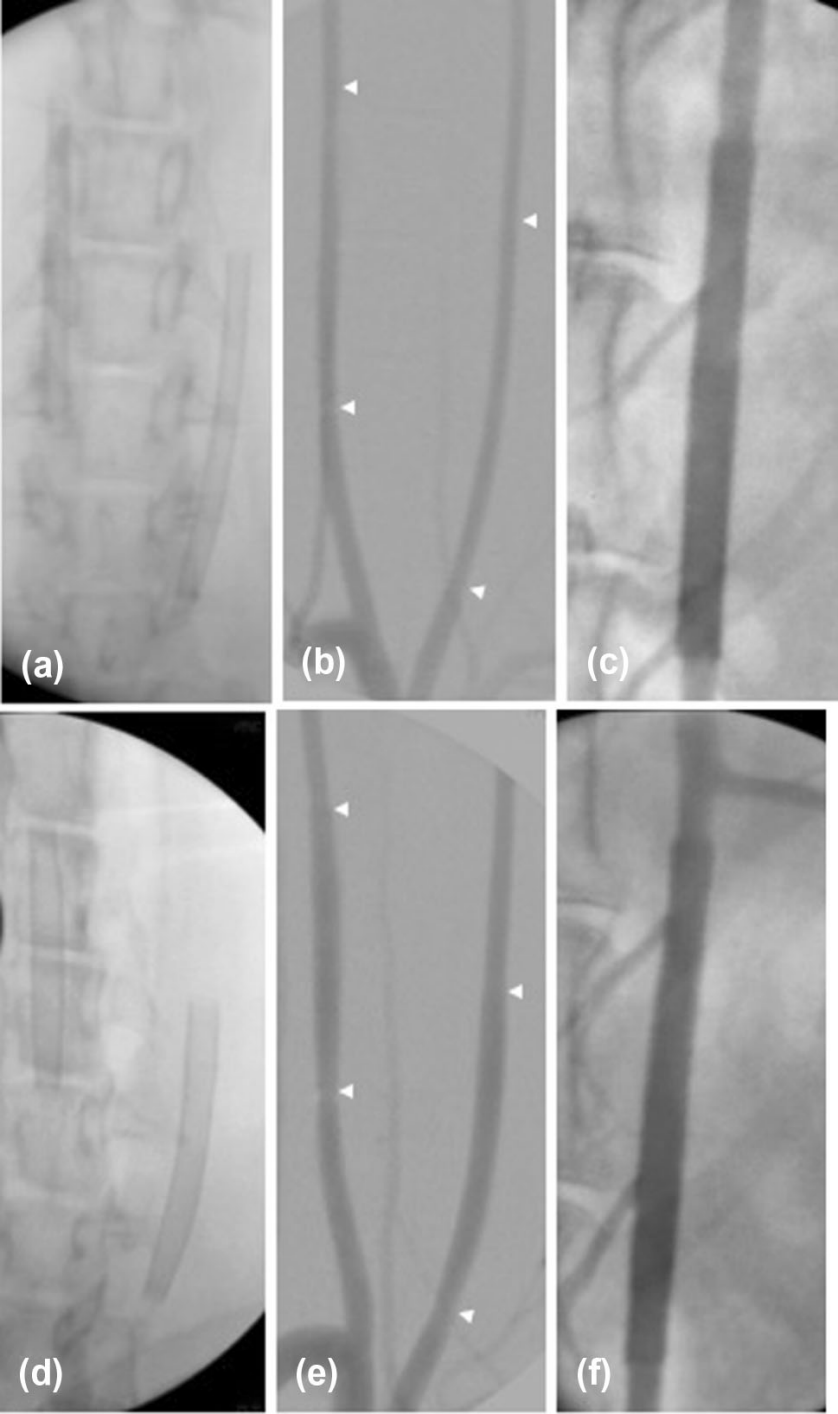
Angiographic imaging of device implantation to measure vessel diameters and stent expansion. Unsubtracted images of the left (Derivo bare) and right (coated Derivo Heal) carotid artery immediately (**a**) and at 28 days (**d**) after flow diverter implantation demonstrating full expansion and good wall apposition of the devices. Digital subtraction angiography of the carotid arteries shows patency of both devices without evidence of in-stent stenosis or thrombosis immediately (**b**) and at 28 days after treatment (**e**). Unsubtracted angiogram of the abdominal artery shows patency of the covered branch arteries immediately (**c**) and at 28 days (**f**) after flow diverter implantation. Proximal and distal device ends are highlighted with white arrow-heads

Angiographic control was performed using a 4 Fr diagnostic catheter before and directly after implantation (Fig. 1a–c) for assessment of vessel diameter and FD expansion. Furthermore, a control angiography was performed prior to FD explantation at the 28-day follow-up (Fig. 1d–f). Selective digital subtraction angiography at the 28-day follow-up was performed via the left femoral artery using a 4 Fr diagnostic catheter (Glidecath) after surgical exposure and introduction of a 4 Fr introducer sheath as described above. The average vessel diameters of the proximal, middle, and distal FD segments were measured by three experienced interventional neuroradiologists (RMB, UY, AS) in consensus on posterior–anterior projection before and after implantation and on day 28. Patency of the branch arteries covered by the devices was also assessed at this follow-up.

### Sample Preparation and Histopathological Processing

Euthanasia by pentobarbital (Narcoren®, Merial GmbH, Hallbergmoos, Germany) overdose (6–8 ml) was performed on day 28 while animals were under anesthesia as described above. The arterial segments with the implanted FDs were surgically removed and fixed in buffered formaldehyde (4%) for at least 48 h and then dehydrated in graded alcohol. For histochemical analyses, samples were pre-infiltrated overnight in ethanol/Technovit® 7200 (1:1) and in pure Technovit 7200 and kept at 4 °C in a desiccator. For embedding, the samples were placed in a small dish, filled with Technovit 7200, and cured with white light using an EXAKT 520 chamber (Norderstedt, Germany) for 10 h, followed by 10 h with UV light. Afterward, the polymerized blocks were cut in slices using a diamond band saw (EXAKT 300/310), followed by grinding and polishing (EXAKT 400CS). The histological slices were stained with hematoxylin and eosin. For each vessel, transversal cuts were made in three different regions (proximal, middle, and central).

### Histology and Histomorphometry

Histological analysis was performed according to different evaluation criteria based on DIN EN ISO 100993–6:2017 and 25,539–2:2013 for biocompatibility studies (Table 1). The evaluation was graded as follows: 0 = none, 1 = minimal, 2 = mild, 3 = moderate, and 4 = intense. Histopathological analysis was conducted by an experienced pathologist (CB) blinded to the coating status of the devices.

**Table 1:** Histopathological evaluation criteria used for grading the vascular healing response and material-tissue reaction

Cellular reaction	Evaluation parameter	Grading
Vascular Healing Response	Endothelium	Endothelialization in % (circumferentially)
	Neointim	FD coverage in % (related to the number of struts)
	Blood clot formation	Semi-quantitative analysis
Material-Tissue Reaction	Neointima thickness	Ratio: Neointima/FD lumen
	FD surface fibrin/platelet deposits	Semi-quantitative analysis
	Inflammation*	Semi-quantitative analysis
	Macrophages	Semi-quantitative analysis
	Calcification	Semi-quantitative analysis

*FD *flow diverter, *plasma cells, lymphocytes, polynuclear cells, and giant cells

After microscopy (VHX-500F; Keyence, Neu-Isenburg, Germany) and digitalization, morphometric measurements were performed for each slice derived from the CCAs and AAs. Maximum and minimum neointimal thickness (NT, defined as the distance between the outer surface of each strut and the luminal border) was measured and the average value was calculated. For the following measurements, the longest and shortest distances between the opposite sides of the lumen profiles were defined as axes. On each axis, the distance between the opposite sides of the lumen profiles and of the FD profile was calculated and defined as distance lumen (*d*_l_) and distance FD (*d*_*s*_). The lumen patency ratio was defined as (*d*_l_/*d*_s_) × 100%. Lumen diameter, FD diameter, and lumen patency ratios were calculated as the averages of *d*_l_, *d*_*s*_, and percentage stenosis ratio on both axes, respectively.

For the semi-quantitative assessment of the material-tissue reaction, at least 10 high power fields were analyzed near the material of each explant and inflammatory cells, macrophages, and potential calcifications were counted. The number of cells was classified according to the ISO norm mentioned above. The platelets and fibrin deposits we analyzed were located around the braid filaments. Furthermore, blood clots were counted around the entire circumference of the blood vessel.

### Statistical Analyses

Statistical analysis was performed using GraphPad Prism (version 7.03, GraphPad Software, San Diego, USA). Data for the two groups were analyzed for normal distribution with the D'Agostino-Pearson normality test. Normally distributed groups were further analyzed with the *t* test, while the not normally distributed groups were analyzed using the Wilcoxon test (paired CCA values) or the Mann–Whitney *U* test (unpaired Aorta values). A two-sided *p* ≤ 0.05 was considered statistically significant.

## Results

### Endovascular Intervention

The mean baseline vessel diameter of the right CCA was 2.2 ± 0.19 mm and the left CCA was 2.3 ± 0.16 mm. The mean vessel diameter of the AA was 3.7 ± 0.25 mm. All devices were implanted successfully. One rabbit carrying a coated FD in the left CCA and non-coated FDs in the AA and right CCA died due to prolonged narcosis after FD implantations. This animal was excluded from the angiographic and histopathological evaluations. In addition, a randomly selected rabbit was excluded from the aortic analysis to compensate for the loss of the deceased animal. We observed one case of a long intraluminal stenosing thrombus in an animal with a Derivo bare in the right CCA at the 28-day follow-up. For the other devices, no in-stent stenosis or thrombosis was observed, neither after implantation nor at the 28-day follow-up. Furthermore, all side branches remained patent throughout the follow-up period. In one case, fish-mouthing of the proximal part of the Derivo bare FD occurred immediately after implantation in the right CCA and appeared aggravated at 28 days. At that time the vessel was occluded.

### Histopathological Analyses

**Fig. 2:**
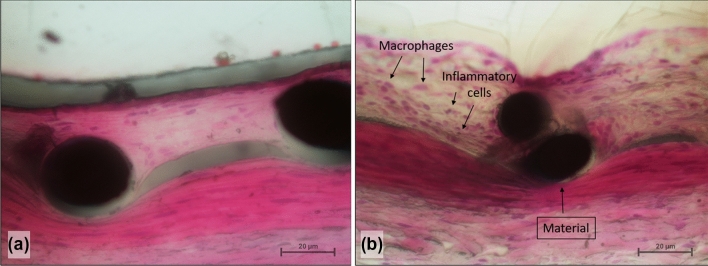
Histopathological imaging to assess immune response. A significantly lower number of macrophages were observed in the coated flow diverters (Derivo Heal) (**a**) compared to uncoated flow diverters (Derivo bare) (**b**) in the common carotid arteries (hematoxylin & eosin staining, 400x)

The histopathological and histometric evaluations were aimed to reveal potential differences in the vessel reactions between both groups, including endothelialization and neointimal thickness. We therefore excluded the two CCAs of the animal with the occluded right CCA from the histopathological analysis. In total, 16 CCA segments (48 slices) and eight AA segments (24 slices) containing the implanted devices were analyzed histologically. The mean endothelialization of both devices showed no significant differences between the bare and the coated embolization devices. The mean fibrin/platelet deposition on the stent struts was rated as minimal with a significantly lower amount of deposition on the Derivo Heal compared to the Derivo bare (*p* = 0.03) in the AA. The mean fibrin/platelet deposition on the neointima was rated none to minimal for both groups in both arterial segments and showed no significant differences. There also was no statistical difference in the number of inflammatory cells between both groups. We detected a significantly lower number of macrophages in the CCAs for the Derivo Heal compared to the Derivo bare (*p* = 0.02) (Fig. 2) but no significant difference in the mean extent of stent coverage. No calcifications were detected in either group. The mean number of blood clots was rated as minimal with no significant differences. The results of the evaluation for the CCA and AA are shown in Tables 2 and 3, respectively.

**Table 2:** Results of the histopathological evaluation of the common carotid artery segments 28 days after implanting either a Derivo bare or Derivo Heal FD

Evaluation criteria for carotid artery	Derivo bare		Derivo heal		*P* value
	Mean	SD	Mmean	SD	
Endothelialization	3.12	0.77	2.92	0.89	0.46
FD surface (fibrin/platelet deposition)	0.50	0.81	0.35	0.63	0.51
Neointima (fibrin/platelet deposition)	0.57	0.11	0.63	0.12	> 0.99
Inflammatory cells	1.23	0.43	1.12	0.33	0.45
Macrophages	2.15	0.73	1.85	0.73	**0.02**
Extent FD coverage	3.85	0.46	3.73	0.83	0.69
Calcifications	0	0	0	0	-
Blood clots	1.12	0.59	0.96	0.53	0.44

*FD* flow diverter; grading (0 = none, 1 = minimal, 2 = mild, 3 = moderate, 4 = severe)

**Table 3:** Results of the histopathological evaluation of the abdominal aorta segments 28 days after implanting either a Derivo bare or Derivo Heal FD

Evaluation criteria for abdominal aorta	Derivo bare		Derivo heal		*P* value
	Mean	SD	Mean	SD	
Endothelialization	2.80	0.63	2.42	0.51	0.17
FD surface (fibrin/platelet deposition)	0.67	0.65	0.17	0.58	0.03
Neointima (fibrin/platelet deposition)	1.42	0.90	0.92	0.29	0.12
Inflammatory cells	1.42	0.51	1.17	0.39	0.37
Macrophages	1.67	0.89	1.50	0.52	0.79
Extent FD coverage	3.17	0.83	3.17	0.94	> 0.99
Calcifications	0.00	0.00	0.00	0.00	–
Blood clots	1.00	0.74	1.00	0.43	> 0.99

*FD* flow diverter; grading (0 = none, 1 = minimal, 2 = mild, 3 = moderate, 4 = severe)

### Histomorphometry

**Fig. 3:**
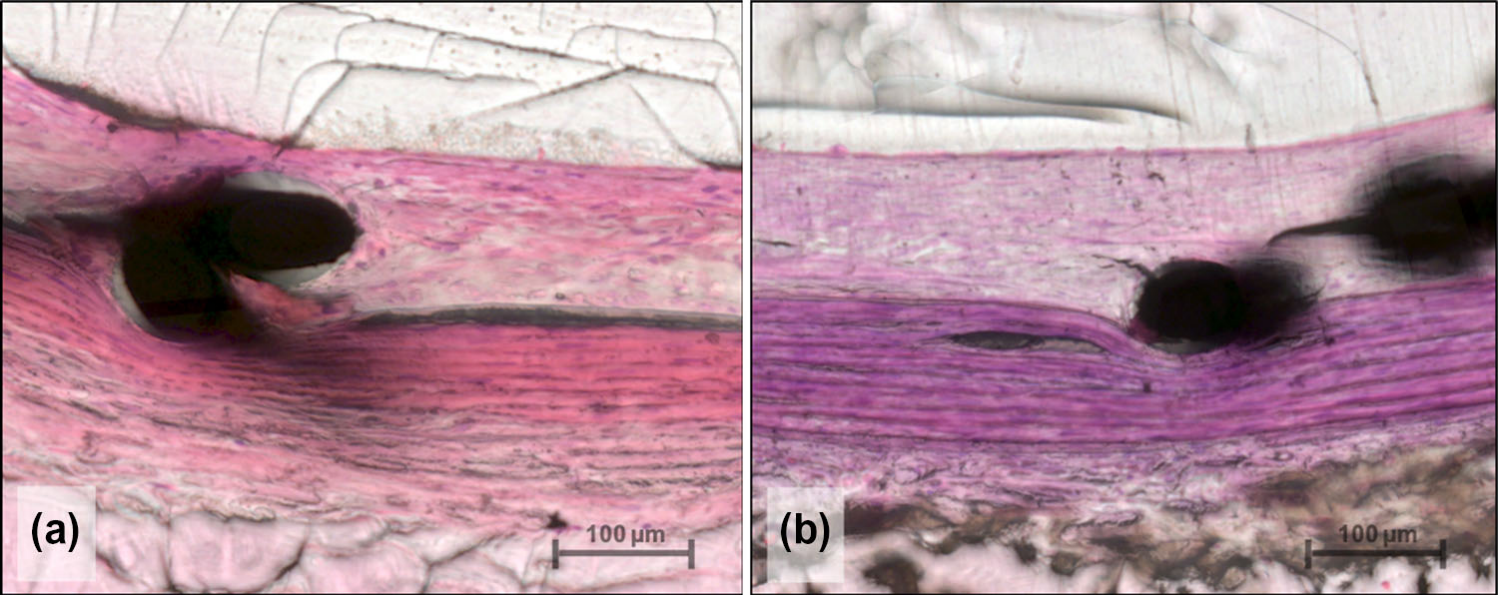
Histomorphometry of the neointima to assess mean maximal thickness and mean stenosis. There were no statistically significant differences in the neointima thickness between the uncoated flow diverters (Derivo bare) (**a**) and the coated flow diverters (Derivo Heal) (**b**) in the common carotid arteries (hematoxylin & eosin staining, 200x)

Measurements of the mean maximal neointima thickness and mean stenosis in the CCA and AA segments yielded no significant differences between the Derivo bare and Derivo Heal groups (Fig. 3 and Table 4).

**Table 4:** Results of the histomorphometric analysis comparing the Derivo bare and Derivo Heal FDs at 28 days post-implantation

Evaluation criteria	Derivo bare		Derivo Heal		*P* value
	Mean	SD	Mean	SD	
Mean maximal neointima thickness CCA	196.6 µm	54.85 µm	194.5 µm	75.06 µm	0.91
Mean maximal neointima thickness AA	176.8 µm	91.83 µm	195.4 µm	43.51 µm	0.59
Mean stenosis CCA	11.7%	11.45%	8.1%	2.9%	0.84
Mean stenosis AA	5.5%	1.7%	5.6%	1.0%	0.87

*CCA* common carotid artery, *AA* abdominal aorta

## Discussion

In the present study, we compared the biocompatibility of coated (Derivo Heal) and non-coated (Derivo bare) flow diverters (FDs) with dual antiplatelet treatment in an animal model. The results demonstrated that both types of FD possess similar levels of biocompatibility. The fibrin-based FD coating did not induce thrombosis angiographically or histologically and had no adverse effects on the rate of re-endothelialization. Known complications after FD treatment such as stent thrombosis and to a lesser extent in-stent stenosis remain limiting factors of this treatment [19]. The present study investigated these limiting factors that have prevented FDs from being used in broader patient populations or in critical situations such as the treatment of acutely ruptured aneurysms [20]. In order to reduce these limitations and extend the field of applications of FDs, great efforts have gone into the development of different coatings for the stent devices, particularly into the development of antithrombogenic heparin coating. However, published results have been rather inconsistent so far. While Tepe et al. [21] did not observe any statistically significant benefit from heparin coating, others could show that heparin coating led to a reduced surface thrombogenicity [22, 23]. Nelson et al. [9] point out the need for optimized heparin molecules to use with endovascular stents. In addition, others have pursued similar surface modification strategies to minimize foreign body reactions and facilitate faster incorporation of the metallic stent surfaces supported by growing neointima [8].

The present study contributes to this ongoing discussion in that it evaluated a novel coating based on a cured artificial fibrin mesh. The performance of this new coating was compared to an uncoated version of the same FD stent type in order to assess potential biocompatibility issues.

Tissue biocompatibility is compulsory for the clinical efficacy of any FD treatment, i.e., it is crucial that the vascular wall heals quickly and incorporates the entire stent into the vessel wall [24]. Furthermore, neither the stent material nor its coating should disturb the overlapping phases of the healing process, namely hemostasis, inflammation, cell proliferation, and remodeling. These orchestrated processes are pivotal to achieve proper healing [25, 26]. Over the 28-day study period, we observed no significant differences in the number of inflammatory cells between either group which indicates that the tested coating elicited a comparable inflammatory response as the bare device. The relatively short follow-up time of 28 days to assess inflammatory processes was chosen to be consistent with previous studies employing rabbit models and explantation at different time points. For instance, in Kallmes et al. [16] neointima thickness and stenosis were maximal one month after implantation and amounted to 200 µm and 20%, respectively, compared to 200 µm and 16% after three months and 100 µm and 15% after six months. Also, Ding et al. [17] observed that the stenosis was maximal at 1-month follow-up in a preclinical study of FD implantation in rabbits. In a previous comparison of FDs with and without coating, implants were explanted after 1 month and showed a mean neointima thickness of 400 µm in both groups [18]. Thus, also in this study the coated material did not induce a higher inflammatory reaction compared to the bare control, which indicates good biocompatibility of the coating used.

Interestingly, we found a significantly lower number of macrophages in the CCA vessel wall with Derivo Heal. It is well known that macrophages play an important role not only in foreign body reactions but also for modulating repair processes involving the M1 and M2 subtype macrophages [27]. In principle, a reduction in macrophages suggests a less severe foreign body reaction, possibly due to the coating covering the stent’s metallic surface. To test this hypothesis, the pathophysiological role of the involved macrophage types would need to be characterized, either as part of the foreign body reaction or as part of the regeneration of the immunohistological typing of the cells. However, this was not possible with the Technovit embedding technique used in the present study.

Based on clinical, radiological, and conventional histological outcomes, and since fibrin-based coatings have been shown to be associated with regenerative processes and the masking of reactive surfaces [28, 29] and also because they have been tested for anti-thrombogenic activity in vitro, [30] we hypothesize that our tested stent material is highly biocompatible. Indeed, our results suggest that the process of cell proliferation and remodeling is not inhibited by the fibrin coating as there was no significant difference in neo-endothelialization. Previous biocompatibility studies have investigated the time course and the spatial distribution of the different cell types within the healing process [31]. Similar studies are needed for fibrin-based coated stents in order to confirm or disprove our hypothesis. In the present study, fibrin/platelet deposition was significantly reduced on the surface of fibrin-based coated FDs compared to bare FDs in the AA but not the CCA. From a pathophysiological point of view, thrombocyte and fibrin deposition should occur during wound healing, i.e., prior to our 28-day follow-up, unless there was a prolonged irritation or a delayed wound healing. Larger in vivo studies are needed to investigate these issues and further clarify the biocompatibility of fibrin-based coated stent devices.

We could confirm that the tested coating has a similar blood and tissue compatibility as non-coated flow diverters. Regarding thrombogenicity due to the tested coating, further studies are needed to evaluate its effect. Since we performed FD implantation under DAPT, we cannot comment on its use with SAPT.

## Study Limitations

Based on our rabbit model and the 28-day post-implantation follow-up, our results appear to be promising and showed good biocompatibility of the newly developed Heal coating. However, more short- and long-term studies are needed to investigate acute and chronic blood and vessel responses to this type of coating, and even longer testing periods are required prior to admitting this coating to clinical practice. These future studies should contain more different time points and should address the topic of endothelialization in more detail. We are currently in the process of preparing a new study that addresses these issues and also includes a higher number of animals.

## Conclusion

In this animal model, a flow diverter coated with a novel fibrin-based coating showed similar blood and tissue compatibility compared to a non-coated flow diverter.
